# Exploiting residue-level and profile-level interface propensities for usage in binding sites prediction of proteins

**DOI:** 10.1186/1471-2105-8-147

**Published:** 2007-05-05

**Authors:** Qiwen Dong, Xiaolong Wang, Lei Lin, Yi Guan

**Affiliations:** 1 School of Computer Science and Technology, Harbin Institute of Technology, Harbin, China

## Abstract

**Background:**

Recognition of binding sites in proteins is a direct computational approach to the characterization of proteins in terms of biological and biochemical function. Residue preferences have been widely used in many studies but the results are often not satisfactory. Although different amino acid compositions among the interaction sites of different complexes have been observed, such differences have not been integrated into the prediction process. Furthermore, the evolution information has not been exploited to achieve a more powerful propensity.

**Result:**

In this study, the residue interface propensities of four kinds of complexes (homo-permanent complexes, homo-transient complexes, hetero-permanent complexes and hetero-transient complexes) are investigated. These propensities, combined with sequence profiles and accessible surface areas, are inputted to the support vector machine for the prediction of protein binding sites. Such propensities are further improved by taking evolutional information into consideration, which results in a class of novel propensities at the profile level, i.e. the binary profiles interface propensities. Experiment is performed on the 1139 non-redundant protein chains. Although different residue interface propensities among different complexes are observed, the improvement of the classifier with residue interface propensities can be negligible in comparison with that without propensities. The binary profile interface propensities can significantly improve the performance of binding sites prediction by about ten percent in term of both precision and recall.

**Conclusion:**

Although there are minor differences among the four kinds of complexes, the residue interface propensities cannot provide efficient discrimination for the complicated interfaces of proteins. The binary profile interface propensities can significantly improve the performance of binding sites prediction of protein, which indicates that the propensities at the profile level are more accurate than those at the residue level.

## Background

Protein function is very often encoded in a small number of residues located in the functional active site, which are dispersed around the primary sequence, but packed in a compact spatial region [1]. Recognition of functional sites in proteins is a direct computational approach to the characterization of proteins in terms of biological and biochemical function. Localization of functional sites will allow us to understand how the protein recognizes other molecules, to gain clues about its likely function at the level of the cell and the organism, and to identify important binding sites that may serve as useful targets for pharmaceutical design [2].

Recently, a series of computational efforts to identify interaction sites or interfaces in proteins have been undertaken. A number of studies on the characteristics of protein interfaces have provided clues for binding site prediction. Several methods have been proposed to predict these sites based on the sequence or structure characteristics of known protein-protein interaction sites.

In terms of physical chemistry, protein interfaces are generally observed to be more hydrophobic than the remainder of the protein surface [3,4]. Moreover, the interfaces of permanent complexes tend to be more hydrophobic when compared to those of transient complexes [5]. Some interfaces have a significant number of polar residues [6], usually where interactions are less permanent [7]. Charged side-chains are often excluded from protein-protein interfaces with the exception of arginine [8], which is one of the most abundant interface residues regardless of interaction types [9]. The evolutionary conservation of residues is another property that may be utilized to predict protein-protein interfaces [10]. The evolutionary trace (ET) method tries to identify functional sites by using the sequence variations and functional divergences found in nature [11,12]. Accurate ET analysis requires functionally relevant sequence and high-quality alignments as input [13]. A structure-independent criterion has been presented to measure the quality of evolutionary trace [14]. Because sequence conservation reflects not only evolutionary selection at binding sites to maintain protein function, but also the selection throughout the protein to maintain the stability of the folded state [15], many researchers try to distinguish functional and structural constraints on protein evolution [16,17]. A comprehensive evaluation of different conservation scores has been performed by Valdar [18]. Other sequence information has also been exploited such as the phylogenetic profile [19,20], the sequence motifs [21], sequence profile [22,23], evolution rate [24,25], etc.

The features extracted from the three-dimensional structures of protein complexes are critical for a full understanding of the mechanism of interactions because they provide specific interaction details at the atomic level. The accessible surface area (ASA) is one of the most widely used features [26]. Molecular docking seems to be the most principled computational approach for identifying the interaction sites [27], but it requires the precise design of energy function [28], either physical energy [29] or empirical scoring functions [27,30]. 3D-motifs have also been successfully used to identify binding sites of the same type in proteins with different folds [31-34]. Patch analysis using a six-parameter scoring function can distinguish the interface from other surfaces [3].

Because none of the above-mentioned properties is able to make an unambiguous identification of interface regions or patches, a combination of some of them (via either a linear combination [35] or machine learning [36]) is found to be effective for improving the accuracy of binding-site prediction [37]. The PINUP method predicts interface residues using an empirical score function made of a linear combination of the energy score, interface propensity and residue conservation score [38].

Rossi et al. first construct a scoring function, and then perform a Monte Carlo optimization, to find a good scoring patch on the protein surface [39].

Machine Learning Methods are well suited to the classification of interface and non-interface surface residues [40,41]. Neural networks [42] and support vector machine [43,44] have been applied in this field. These studies take sequential or structural information as input [6]. Other researchers adopt two-stage model [23] to further improve the performance. Recently, the conditional random field (CRF) model has been introduced, which formalizes the prediction of protein interaction sites as a sequence-labeling task [45].

In this study, we revisit the difference of amino acid compositions between the interface area and other surface area. Although some researchers have found that there are different amino acid compositions among the interaction sites of different complexes (homo-permanent complexes, homo-transient complexes, hetero-permanent complexes, and hetero-transient complexes) [46], such difference has not been integrated into the prediction process. Here, the residue interface propensities of different complexes are collected. These propensities, combined with sequence profiles and accessible surface areas, are inputted to the support vector machine for the prediction of protein binding sites. Such propensities are further improved by taking evolutional information into consideration. The frequency profiles are directly calculated from the multiple sequence alignments outputted by PSI-BLAST [47] and converted into binary profiles [48] with a probability threshold. As a result, the protein sequences are represented as sequences of binary profiles rather than sequences of amino acids. Similar to the residue interface propensities, a class of novel propensities at the profile level is introduced. Binary profiles can be viewed as novel building blocks of proteins. It has been successfully applied in many computational biology tasks, such as domain boundary prediction [48], knowledge-based mean force potentials [49], protein remote homology detection [50] etc. Experimental results show that the binary profile propensities significantly improve the performance of binding sites prediction of proteins.

## Results and discussion

### Residue interface propensities

**Figure 1:**
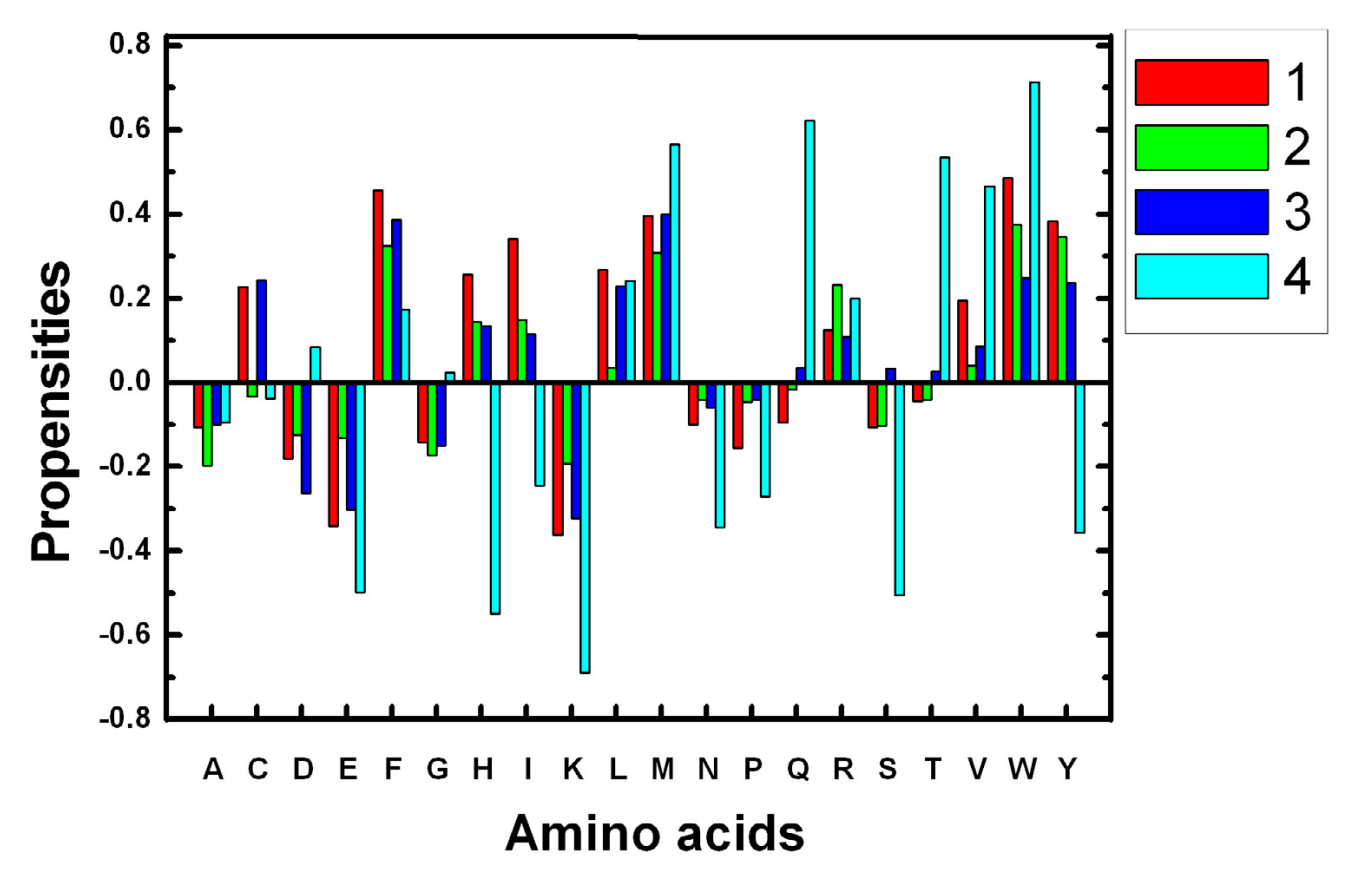
**Residue interface propensities of the four kinds of complexes**. Column bar 1, 2, 3 and 4 denote hetero-permanent, hetero-transient, homo-permanent and homo-transient complex respectively.

Residue interface propensities are good indicators for binding sites and have been widely used in many studies [6]. The residue interface propensities of the four kinds of complexes are shown in Fig. 1. Positive propensity means that the residue is abundant in the interface while negative propensity means that the residue is abundant in the surface area.

The four kinds of complexes have similar residue interface propensities. They all show that hydrophobic residues (F, I, L, M, V) and some polar aromatic residues (W, Y, H) are favored in interface area. The charged residue R also shows preferences for the interface area. Other polar amino acid T, E and small amino acid P, A are disfavored in the interface. The same phenomena have been observed by others [35] although some researchers evaluated the ASA contribution for amino acid [3,38] while we count them. Bio-physically similar residues, such as L and I, or D and E, usually showed similar trends, indicating the reliability of the data.

There are minor differences among the four kinds of complexes. Although many amino acids show the same trend for interface area or surface area, the propensities are different for the four kinds of complexes. Further more, some amino acids reveal different propensities in different complexes. Amino acid Q, S and T show preferences for the hetero complexes rather than homo complexes.

Amino acid C and L are favored in permanent complexes rather than transient complexes. Ofran and Rost [46] found that the composition of all interface types differed substantially from that of SWISS-PROT. Here we conclude that the residue interface propensities show general trends and have minor differences among different kinds of complexes.

### Binary profile interface propensities

The binary profile frequencies in interface are different from those in surface area. These differences can be used to produce the discriminative binary profile propensities. In theory, the total number of binary profiles is extremely large (2^20^), but in fact, only a small fraction of binary profiles appears, which is dependent on the choice of probability threshold *P*_*h *_and the dataset. Based on the results of cross-validation (Next section), the four kinds of complexes have different number of binary profiles, ranging from one hundred to several thousands. The binary profiles and their propensities of the four kinds of complexes are listed in the Additional files (see additional file 1, 2, 3, 4). Note that the binary profiles with low occurrence times (<3) are ignored, since these profiles are not statistically significant and may introduce much noise.

An increased propensity of hydrophobic residues and their combinations in interface has been observed, such as the binary profile FHWY, ILMV. Although some amino acids are preferred in surface, the combination of these amino acids with other amino acids may be preferred in interface such as AEP, ST. Another special phenomenon is that some binary profiles only occur in interface while other binary profiles only occur in surface area. The former results in a maximum propensity (being set as 4) and the latter results in a minimum propensity (being set as – 4). Each kind of complexes has many such binary profiles.

The differences of binary profile interface propensities among different complexes are significant in comparison with those of residue interface propensities. Many binary profiles show positive propensities in one complex but negative propensities in another complex. Table 1 summarizes the number of such binary profiles between any pair of complexes.

**Table 1:** The differences of binary profile interface propensities among the four kinds of complexes

	Hetero permanent^a^	Hetero transient	Homo permanent	Homo transient
Hetero permanent	-	341	378	29
Hetero transient	261	-	893	28
Homo permanent	267	908	-	36
Homo transient	17	27	38	-

^a^Given in the element (I, J) of the matrix are the number of binary profiles which show positive propensities in complex type I and negative propensities in complex type J.

### Comparative results with and without propensities

The first SVM takes profile and ASA of spatially neighboring residues as input, which are common input features used by previous studies [15,44,51]. Then we add the amino acid or binary profile interface propensities as an extra feature to evaluate whether these propensities can improve the performance or not. All the results are obtained by five-fold cross-validation.

The second SVM takes residue interface propensities as an extra feature. Table 2 gives the results with and without residue interface propensities. The similar performance indicates that the standard amino acid cannot provide efficient discrimination for the complicated interfaces of proteins. The results on homo-transient complex are extremely low because there are only 5 chains in this complex. The performance of the first SVM is comparable with those of Chung et al. [15]. They reported precision of 0.498 and recall of 0.568 with the same features on their 274 hetero-complexes.

**Table 2:** Comparative results with and without residue interface propensities on the four kinds of complexes.

		Precision	Recall	F1	Accuracy	CC
Hetero permanent	Non-pro^a^	0.518	0.582	0.547	0.687	0.267
	AA-pro^b^	0.514	0.590	0.548	0.684	0.265
Hetero transient	Non-pro	0.414	0.563	0.475	0.643	0.204
	AA-pro	0.415	0.561	0.476	0.643	0.204
Homo permanent	Non-pro	0.463	0.607	0.526	0.687	0.288
	AA-pro	0.474	0.617	0.536	0.693	0.303
Homo transient	Non-pro	0.206	0.463	0.279	0.691	0.136
	AA-pro	0.260	0.465	0.327	0.743	0.195

^a^The features of SVM are Position-Specific Score Matrix (PSSM) and Accessible Surface Areas (ASA)

^b^Residue interface propensity is inputted to SVM as an extra feature

**Figure 2:**
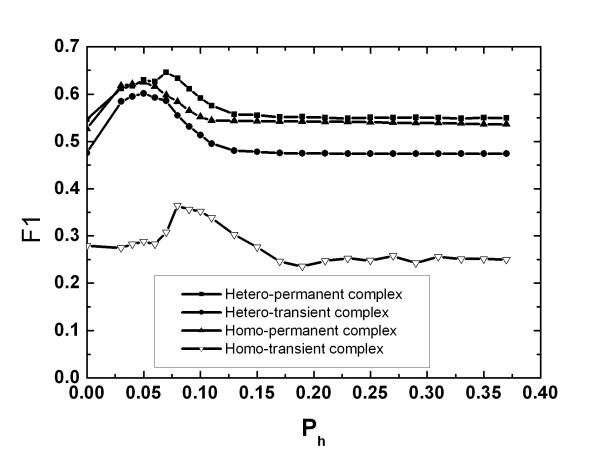
**The average F1 under different value of parameter Ph**. The F1 is obtained as the results of cross-validation at the validation dataset.

The third SVM takes binary profile interface propensities as an extra feature instead of residue interface propensities. The probability threshold *P*_*h *_of converting a frequency profile into a binary profile needs to be optimized. During the validation process, three sets are used to train SVM, one validation set is used to optimize the parameter and the testing set is used to give the final results. That is, we select the values of *P*_*h *_that give the best results on the validation set and then such parameter is used to test the proteins on the testing set to give the final results. The influences of *P*_*h *_on the performance are illustrated in Fig. 2. F1 is used as the guild line since it is a tradeoff between precision and recall. The optimal values of *P*_*h *_are different for different complexes.

The results of cross-validation are then obtained with the optimal value of *P*_*h *_and shown in Table 3. The improvement of the third SVM is significant in comparison with the other two SVMs. The F1 is improved by about ten percent. According to the experimental results, we can infer that the propensities at the profile level may be more accurate than that at the amino acid level.

**Table 3:** Cross-validation results with binary profile interface propensities

	*P*_*h*_^a^	N^b^	Precision	Recall	F1	Accuracy	CC
Hetero permanent	0.07	1558	0.599	0.700	0.644	0.735	0.396
Hetero transient	0.05	4662	0.501	0.756	0.602	0.697	0.379
Homo permanent	0.05	8639	0.546	0.734	0.626	0.745	0.435
Homo transient	0.08	129	0.277	0.551	0.363	0.747	0.250

^a^The optimal probability threshold *P*_*h *_of converting a frequency profile into a binary profile

^b^The number of binary profiles

### Comparative results with propensities from other complexes

Analysis of interface propensities shows that the residue interface propensities have minor differences among different complexes while the profile interface propensities differ significantly among different complexes. To validate it, the propensities from other complexes are used as an extra feature. The results are shown in Table 4 (residue-level) and Table 5 (profile-level).

**Table 4:** Comparative results with residue interface propensities from other complexes.

Complex^a^	Propensities^b^	Precision	Recall	F1	Accuracy	CC
Hetero permanent	Hetero transient	0.512	0.570	0.539	0.679	0.256
	Homo permanent	0.503	0.578	0.538	0.682	0.250
Hetero transient	Hetero permanent	0.419	0.568	0.482	0.646	0.212
	Homo permanent	0.418	0.568	0.482	0.644	0.210
Homo permanent	Hetero permanent	0.445	0.596	0.510	0.674	0.273
Homo transient	Hetero permanent	0.192	0.550	0.285	0.636	0.132

^a^The complexes that the experiments are performed on.

^b^The complexes that the propensities are derived from.

**Table 5:** Comparative results with binary profile interface propensities from other complexes.

Complex^a^	Propensities^b^	Precision	Recall	F1	Accuracy	CC
Hetero permanent	Hetero transient	0.532	0.574	0.551	0.698	0.282
Hetero transient	Homo permanent	0.413	0.562	0.475	0.642	0.203
Homo permanent	Hetero Permanent	0.463	0.607	0.525	0.686	0.287
Homo transient	Hetero permanent	0.181	0.514	0.262	0.637	0.111

^a^The complexes that the experiments are performed on.

^b^The complexes that the propensities are derived from.

The performances of Table 4 are close to those of Table 2, which indicates that the differences of residue interface propensities among different complexes can be negligible. The performances of Table 5 decrease significantly in comparison with those of Table 3, so the profile interface propensities are sensitive to the types of complexes. In other words, the propensities at the profile-level can give more exact description of interfaces than the propensities at the residue level.

### Examples

**Figure 3:**
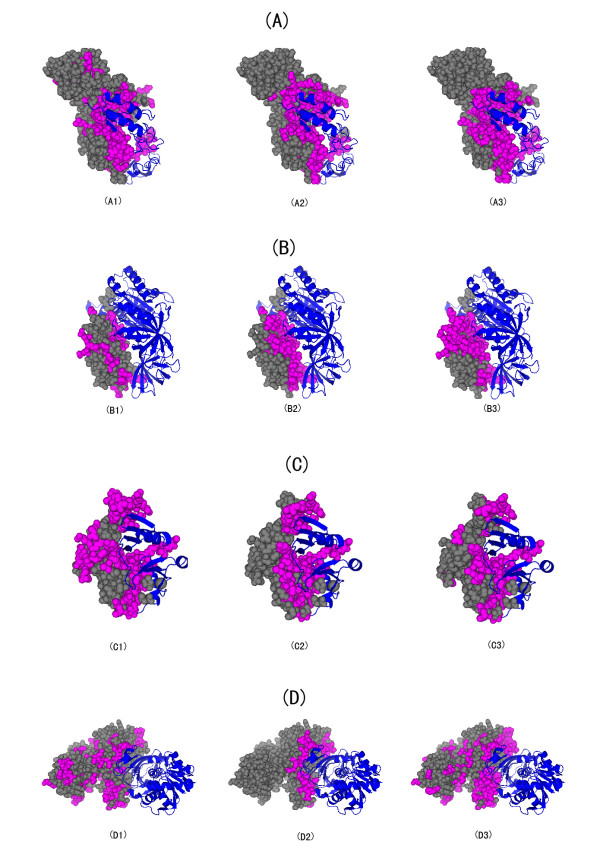
**Sample predictions**. One protein is selected from each complexes and shown in sub-figure (A), (B), (C) and (D). The PDB IDs and chain identifiers are 1bplB, 1ijeB, 1lqpB and 1j0xO respectively. The interface residues are depicted with purple colour. For each sub-figure, the true interfaces are shown in the center picture. The left picture gives the results predicted by the second SVM, which takes residue interface propensities as an extra feature. The right picture gives the results predicted by the third SVM, which takes binary profile propensities as an extra feature.

Some examples are provided at Fig. 3. One protein is selected from each type of complexes. The true interface and the interface predicted by the second SVM and the third SVM are depicted. Most interface residues and non-interface residues can be predicted correctly. It is clearly that the classifier that integrates binary profile interface propensities is more accurate than the classifier that uses residue interface propensities.

### Comparison with conservation scores

The conservation score is another widely used feature in prediction of function sites, which indicates the importance of a residue for maintaining the structure and function of a protein [18]. Here, we compare the binary profile interface propensities with conservation scores since both of them are derived from the multiple sequence alignment of homologues. Three conservation scores are investigated including the symbol entropy score [52], Karlin score [53] and Valdar score [54]. They are defined as follows:

Centropy=−∑iKpiln⁡pi×1ln⁡K
 MathType@MTEF@5@5@+=feaafiart1ev1aaatCvAUfKttLearuWrP9MDH5MBPbIqV92AaeXatLxBI9gBaebbnrfifHhDYfgasaacH8akY=wiFfYdH8Gipec8Eeeu0xXdbba9frFj0=OqFfea0dXdd9vqai=hGuQ8kuc9pgc9s8qqaq=dirpe0xb9q8qiLsFr0=vr0=vr0dc8meaabaqaciaacaGaaeqabaqabeGadaaakeaacqWGdbWqdaWgaaWcbaGaemyzauMaemOBa4MaemiDaqNaemOCaiNaem4Ba8MaemiCaaNaemyEaKhabeaakiabg2da9iabgkHiTmaaqahabaGaemiCaa3aaSbaaSqaaiabdMgaPbqabaGccyGGSbaBcqGGUbGBcqWGWbaCdaWgaaWcbaGaemyAaKgabeaakiabgEna0oaalaaabaGaeGymaedabaGagiiBaWMaeiOBa4Maem4saSeaaaWcbaGaemyAaKgabaGaem4saSeaniabggHiLdaaaa@4E37@

CKarlin=∑iN∑j>iNM(si,sj)×2N(N−1)
 MathType@MTEF@5@5@+=feaafiart1ev1aaatCvAUfKttLearuWrP9MDH5MBPbIqV92AaeXatLxBI9gBaebbnrfifHhDYfgasaacH8akY=wiFfYdH8Gipec8Eeeu0xXdbba9frFj0=OqFfea0dXdd9vqai=hGuQ8kuc9pgc9s8qqaq=dirpe0xb9q8qiLsFr0=vr0=vr0dc8meaabaqaciaacaGaaeqabaqabeGadaaakeaacqWGdbWqdaWgaaWcbaGaem4saSKaemyyaeMaemOCaiNaemiBaWMaemyAaKMaemOBa4gabeaakiabg2da9maaqahabaWaaabCaeaacqWGnbqtcqGGOaakcqWGZbWCdaWgaaWcbaGaemyAaKgabeaakiabcYcaSiabdohaZnaaBaaaleaacqWGQbGAaeqaaOGaeiykaKIaey41aq7aaSaaaeaacqaIYaGmaeaacqWGobGtcqGGOaakcqWGobGtcqGHsislcqaIXaqmcqGGPaqkaaaaleaacqWGQbGAcqGH+aGpcqWGPbqAaeaacqWGobGta0GaeyyeIuoaaSqaaiabdMgaPbqaaiabd6eaobqdcqGHris5aaaa@5581@

Cvaldar=λ∑iN∑j>iNwiwjM(si,sj)
 MathType@MTEF@5@5@+=feaafiart1ev1aaatCvAUfKttLearuWrP9MDH5MBPbIqV92AaeXatLxBI9gBaebbnrfifHhDYfgasaacH8akY=wiFfYdH8Gipec8Eeeu0xXdbba9frFj0=OqFfea0dXdd9vqai=hGuQ8kuc9pgc9s8qqaq=dirpe0xb9q8qiLsFr0=vr0=vr0dc8meaabaqaciaacaGaaeqabaqabeGadaaakeaacqWGdbWqdaWgaaWcbaGaemODayNaemyyaeMaemiBaWMaemizaqMaemyyaeMaemOCaihabeaakiabg2da9GGaciab=T7aSnaaqahabaWaaabCaeaacqWG3bWDdaWgaaWcbaGaemyAaKgabeaakiabdEha3naaBaaaleaacqWGQbGAaeqaaaqaaiabdQgaQjabg6da+iabdMgaPbqaaiabd6eaobqdcqGHris5aaWcbaGaemyAaKgabaGaemOta4eaniabggHiLdGccqWGnbqtcqGGOaakcqWGZbWCdaWgaaWcbaGaemyAaKgabeaakiabcYcaSiabdohaZnaaBaaaleaacqWGQbGAaeqaaOGaeiykaKcaaa@5483@

Please refer to [18] for detail calculation and comparison of these scores.

These conservation scores are used as an additional feature respectively and the cross-validation results are shown in Table 6. Overall the F1 is improved by about 2 percent in comparison with those without conservation scores (the first SVM).

**Table 6:** Cross-validation results with conservation scores

		Precision	Recall	F1	Accuracy	CC
Hetero permanent	V_entropy_	0.529	0.571	0.549	0.692	0.280
	V_Karlin_	0.531	0.584	0.556	0.698	0.282
	V_Valdar_	0.534	0.592	0.561	0.702	0.283
Hetero transient	V_entropy_	0.414	0.563	0.477	0.644	0.203
	V_Karlin_	0.414	0.572	0.480	0.644	0.204
	V_Valdar_	0.415	0.585	0.486	0.645	0.205
Homo permanent	V_entropy_	0.464	0.607	0.526	0.687	0.288
	V_Karlin_	0.472	0.613	0.533	0.692	0.291
	V_Valdar_	0.478	0.622	0.541	0.698	0.295
Homo transient	V_entropy_	0.212	0.468	0.292	0.698	0.121
	V_Karlin_	0.226	0.478	0.307	0.710	0.127
	V_Valdar_	0.228	0.482	0.310	0.721	0.132

All these conservation scores show positive correlation with binary profile interface propensities, although the Pearson correlation coefficients are small (0.017, 0.053, 0.064 for V_entropy_, V_Karlin _and V_valdar _respectively). The results show that the improvement by conservation scores is much lower than that by binary profile interface propensities.

### Independent testing

A direct comparison with other studies is difficult due to the differences in choice of dataset and definitions of surface or interface residue. Our method is tested on the protein-protein docking benchmark 2.0, which is a well established dataset including 84 hetero transient complex. The proteins in hetero transient complexes are filtered by removing the protein chains contained in benchmark 2.0 dataset and their homologues. The SVMs are re-trained on the filtered datasets and used to test the complexes in benchmark 2.0 dataset. The results on different subset (rigid-body, medium difficult and difficult set) and the average results are shown in Table 7. The classifiers with binary profile interface propensities outperform those with residue interface propensities by 5 percent in term of F1.

**Table 7:** Results on the protein-protein docking benchmark 2.0 dataset.

Subset	No. of Protein	Method^a^	Precision	Recall	F1	Accuracy	CC
Rigid body	63	AA	0.393	0.447	0.418	0.848	0.301
		BP	0.446	0.495	0.469	0.857	0.328
Medium difficult	13	AA	0.356	0.405	0.379	0.810	0.258
		BP	0.412	0.464	0.436	0.821	0.271
Difficult	8	AA	0.362	0.384	0.372	0.813	0.299
		BP	0.409	0.427	0.428	0.819	0.317
All	84	AA	0.370	0.412	0.390	0.824	0.286
		BP	0.422	0.462	0.441	0.832	0.305

^a^AA, the classifiers with residue interface propensities as extra features; BP, the classifiers with binary profile interface propensities as extra features.

The results are better than those of related works. Liang et al [38]. developed an empirical scoring function for binding site prediction, which is a weighted combination of energy scores, conservation scores and residue interface propensities. They achieved the precision of 0.294 and the recall of 0.305. The overall F1 is only 0.30. Their method is trained on a small dataset (only 57 proteins). Furthermore their method is a simple combination of three features while our method is based on discriminative model.

## Conclusion

In this study, the residue interface propensities of four kinds of complexes (hetero-permanent complex, hetero-transient complex, homo-permanent complexes and homo-transient complex) are collected and applied in the process of predicting binding sites of proteins. Such propensities are improved by taking evolutionary information into consideration, which results in the binary profile interface propensities. Although there are minor differences among the four kinds of complexes, the residue interface propensities cannot provide efficient discrimination for the complicated interfaces of proteins. The binary profile interface propensities can significantly improve the performance of binding sites prediction of protein, which indicates that the propensities at the profile level are more accurate than those at the residue level.

## Methods

### Dataset

A comprehensive set of complexes is chosen from the Protein Data Bank (PDB) [55] and then subjected to a number of stringent filtering steps. All proteins with multi-chains, non-NMR structures and resolution better than 4 Å are selected. Two chains in a protein are considered as interacting pairs if at least two non-hydrogen atoms in each chain are separated by no more than 5 Å [42,56].

For PDB structure with more than two chains, each chain is selected for at most one time. For protein chain that interacts with multiple partners, only one partner with the most interfacial residues is selected as its partner. The protein chains with less than 40 amino acids are removed. The PQS web-server [57] is used to eliminate crystal packing complexes rather than biologically functional multimers. The selected chains are further filtered such that no pair of chain has more than 25% sequence identify. Finally, a total of 1139 chains are obtained.

### Classification of complexes

The protein-protein interactions can be divided into different types according to different criterions [58]. In this study, the complexes are classified by the homology of interacting chains (homo versus hetero) and the lifetime of the complexes (transient versus permanent).

Using simple sequence comparisons, the complexes can be classified as homo-complexes or hetero-complexes. Two interacting protein chains were defined as homo-complex if over 90% of them are aligned and the sequence identity over the aligned region is more than 95% [42]. All other complexes are classified as hetero-complexes.

A permanent complex is usually very stable and thus only exists in its complexed form. In contrast, a transient complex can exist in separated state. The method of differentiating the transient complexes and permanent complexes is same as the one used by Ofran and Rost [46]. The guild lines for classifying the hetero-complexes and homo-complexes into permanent and transient states are different. They are briefly described here. If the chains from the hetero-complexes are stored in the same SWISS-PROT files [59], the complexes are classified as hetero-permanent complexes; otherwise they are classified as hetero-transient complexes. All homo-complexes that are annotated as monomers in DIP [60] database are classified as homo-transient complexes; otherwise they are classified as homo-permanent complexes.

The above dataset is then grouped into four kinds of complexes (hetero-permanent, hetero-transient, homo-permanent, homo-transient). The statistical information of different complexes is tabulated in Table 8. An amino acid is defined as a surface amino acid if the ASA of at least one of its atom is larger than 2 Å^2 ^[39]. A surface residue is considered as interface residues if its accessible surface area is decreased by more than 1 Å^2 ^upon complexation [38]. The ASA is calculated with the DSSP program [61]. According to this definition, 27.3% of the surface residues are interface residues. Such ratio is very close to that (28%) in Chung's dataset [15].

**Table 8:** Summary of the four complexes

	Chains	Res.	Surface res.	Interface res.^a^
Hetero-permanent	123	25157	21737	7136 (32.8%)
Hetero-transient	386	86168	72288	19177 (26.5%)
Homo-permanent	625	174629	142620	38556 (27%)
Homo-transient	5	1555	1267	187 (14.8%))
Total	1139	287509	237912	65056 (27.3%)

^a^Given in the bracket are the fraction of interface residues in the total number of surface residues.

### Calculation of propensities

The amino acid frequencies between interface and other surface area are different. Such difference can be used to produce the residue interface propensity, which is defined as the log ratio between the amino acid frequency in interface area and that in surface area:

*P*_*a *_= In(*P*_*a*, *I*_/*P*_*a*, *S*_)

where *P*_*a *_is the propensity of amino acid *a*, *P*_*a*, *I *_is the frequency of amino acid *a *in interface area and *P*_*a*, *S *_is the frequency of amino acid *a *in surface area. The frequencies can be calculated from the training set by maximum likelihood estimation:

Pa,I=Ca,ICI
 MathType@MTEF@5@5@+=feaafiart1ev1aaatCvAUfKttLearuWrP9MDH5MBPbIqV92AaeXatLxBI9gBaebbnrfifHhDYfgasaacH8akY=wiFfYdH8Gipec8Eeeu0xXdbba9frFj0=OqFfea0dXdd9vqai=hGuQ8kuc9pgc9s8qqaq=dirpe0xb9q8qiLsFr0=vr0=vr0dc8meaabaqaciaacaGaaeqabaqabeGadaaakeaacqWGqbaudaWgaaWcbaGaemyyaeMaeiilaWIaemysaKeabeaakiabg2da9maalaaabaGaem4qam0aaSbaaSqaaiabdggaHjabcYcaSiabdMeajbqabaaakeaacqWGdbWqdaWgaaWcbaGaemysaKeabeaaaaaaaa@3948@

Pa,S=Ca,SCS
 MathType@MTEF@5@5@+=feaafiart1ev1aaatCvAUfKttLearuWrP9MDH5MBPbIqV92AaeXatLxBI9gBaebbnrfifHhDYfgasaacH8akY=wiFfYdH8Gipec8Eeeu0xXdbba9frFj0=OqFfea0dXdd9vqai=hGuQ8kuc9pgc9s8qqaq=dirpe0xb9q8qiLsFr0=vr0=vr0dc8meaabaqaciaacaGaaeqabaqabeGadaaakeaacqWGqbaudaWgaaWcbaGaemyyaeMaeiilaWIaem4uamfabeaakiabg2da9maalaaabaGaem4qam0aaSbaaSqaaiabdggaHjabcYcaSiabdofatbqabaaakeaacqWGdbWqdaWgaaWcbaGaem4uamfabeaaaaaaaa@3984@

where *C*_*a*, *I *_is the count of amino acid *a *in interface area, *C*_*I *_is the total number of amino acid in interface area, *C*_*a*, *S *_is the count of amino acid *a *in surface area, *C*_*S *_is the total number of amino acid in surface area. The residue interface propensity describes the likelihood of amino acid to be found in interface area as compared to those in surface area. A propensity of 0 indicates that the amino acid has the same frequency in interface and surface area. A positive propensity means that the amino acid is over-representative in interface area.

In term of binary profile, the protein sequence is represented as sequence of binary profiles rather than sequence of amino acids. Each amino acid is replaced by the corresponding binary profiles that are derived from the multiple sequence alignments as described in the following section. The calculation formula of binary profile interface propensities are same as that of the residue interface propensities except that the subscripts are replaced by binary profiles rather than amino acid:

*P*_*b *_= In(*P*_*b*, *I*_/*P*_*b*, *S*_)

where *P*_*b *_is the propensity of binary profile *b*, *P*_*b*, *I *_is the frequency of binary profile *b *in interface area and *P*_*b*, *S *_is the frequency of binary profile *b *in surface area. The frequencies can also be calculated by maximum likelihood estimation in the same manner of amino acid. The binary profile interface propensity contains evolution information and provides more accurate prediction of binding sites than amino acid interface propensity according to the experimental results.

Here an example of calculating the propensities of binary profiles is provided. Suppose there is a frequency profile (see Table 9):

**Table 9:** An example of calculating the propensities of binary profiles

**A: 0.09**	C: 0.02	D: 0.07	E: 0.04	F: 0.03	**G: 0.1**	H: 0.07	I: 0.04	K: 0.02	**L: 0.09**
M: 0.02	**N: 0.09**	P: 0.05	Q: 0.04	R: 0.03	S: 0.04	T: 0.05	V: 0.01	W: 0.05	Y: 0.05

When the probability threshold *P*_*h *_is taken as 0.08, we get the following binary profile (see Table 10):

**Table 10:** When the probability threshold *P_h_* is taken as 0.08, we get the following binary profile:

**A: 1**	C: 0	D: 0	E: 0	F: 0	**G: 1**	H: 0	I: 0	K: 0	**L: 1**
M: 0	**N: 1**	P: 0	Q: 0	R: 0	S: 0	T: 0	V: 0	W: 0	Y: 0

By collecting the non-zero term in binary profile, the combination of amino acid AGLN is obtained. Suppose the frequency of AGLN is 0.00042 in interface area and 0.00021 in surface area, which are calculated by maximum likelihood estimate using equation (5) and (6). Thus, the propensity of AGLN is 0.693147 (ln (0.00042/0.00021)) by equation (7).

### Generating of binary profiles

A binary profile can be expressed by a vector with dimensions of 20, in which each element represents one kind of amino acid and can only take value of 0 or 1. When the element takes value of 1, it means that the corresponding amino acid can occur during evolution. Otherwise, it means that the corresponding amino acid cannot occur. A binary profile can also be expressed by a substring of amino acid combination, which is obtained by collecting each element of the vector with non-zero value. Each combination of the twenty amino acids corresponds to a binary profile and vice versa. Below we describe the process of generating the binary profiles.

The PSI-BLAST [47] is used to generate the profiles of amino acid sequences with parameters j = 3 and e = 0.001. The search is performed against the non-redundant database (NR) database from NCBI. The frequency profiles are directly obtained from the multiple sequence alignments outputted by PSI-BLAST. The target frequency reflects the probability of an amino acid occurrence in a given position of the sequences. The method of target frequency calculation is similar to that implemented in PSI-BLAST.

**Figure 4:**
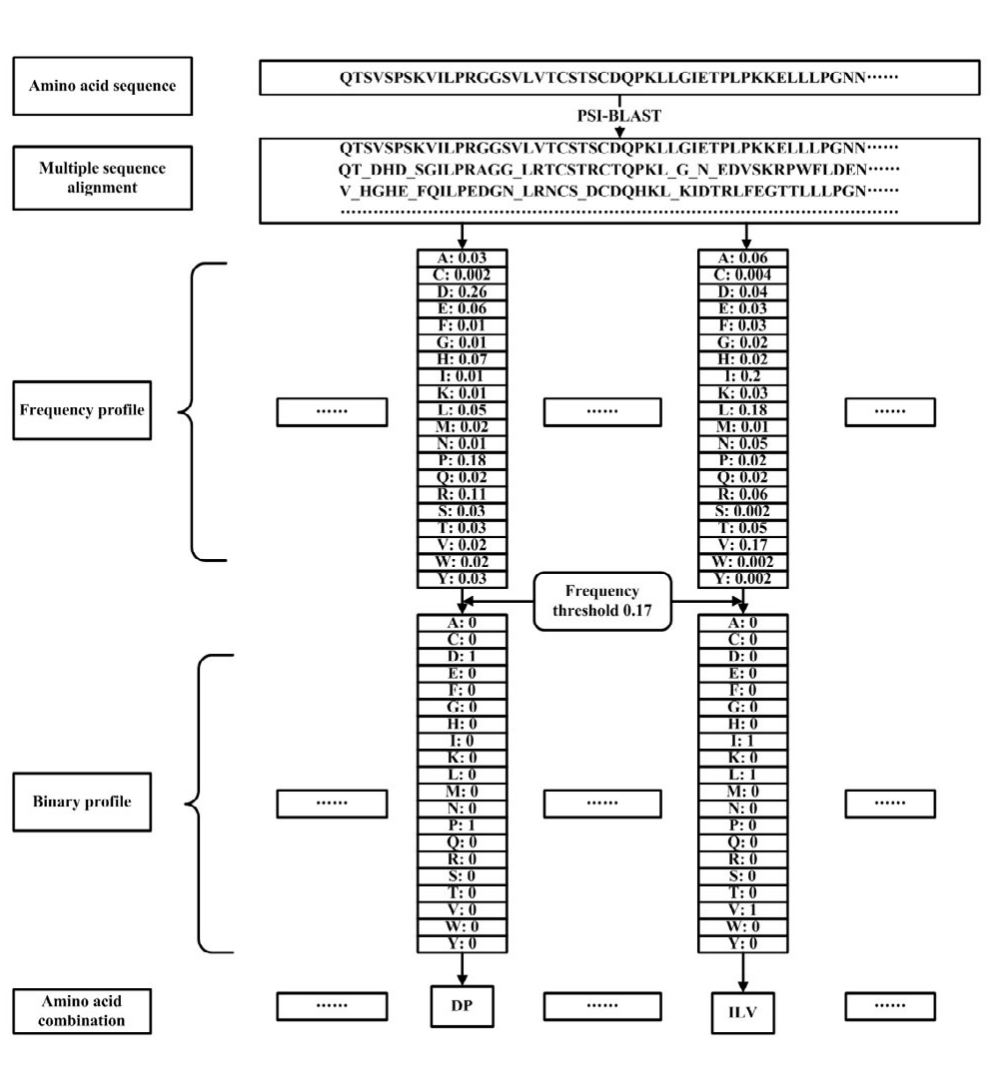
**The flowchart of generating binary profiles**. The multiple sequence alignment is obtained by PSI-BLAST. The frequency profile is calculated from the multiple sequence alignment and converted to a binary profile with a frequency threshold. The substring of amino acid combination is then collected.

Because the frequency profile is a matrix of frequencies for all amino acids, it cannot be directly used and need to be converted into a binary profile by a probability threshold *P*_*h*_. When the frequency of an amino acid is larger than *P*_*h*_, it is converted into an integral value of 1, which means that the specified amino acid can occur in a given position of the protein sequence during evolution. Otherwise it is converted into 0. A substring of amino acid combination is then obtained by collecting the binary profile with non-zero value for each position of the protein sequences. These substrings have approximately represented the amino acids that possibly occur at a given sequence position during evolution. Fig. 4 has shown the process of generating binary profiles.

### Prediction

Support Vector Machine (SVM) is a class of supervised learning algorithms first introduced by Vapnik [62]. Given a set of labelled training vectors (positive and negative input examples), SVM can learn a linear decision boundary to discriminate between the two classes. The result is a linear classification rule that can be used to classify new test examples. SVM has exhibited excellent performance in practice and has strong theoretical foundation of statistical learning theory. Here the LIBSVM package [63] is used as the SVM implementation with radial basis function as kernel. The values of γ and regularization parameter C are set to be 0.005 and 1, respectively.

The input of SVM is a window containing a surface residue and its 12 spatially nearest surface residues [15]. An interface residue is defined as the positive sample, and a surface residue is defined as the negative sample. The input features are sequence profiles, accessible surface areas and propensities of residues in the window. The sequence profiles are taken from the Position-Specific Score Matrix (PSSM) outputted by PSI-BLAST [47]. All the input values are scaled between -1 and 1 before being inputted to the SVM.

It is known that SVM cannot perform well on an unbalanced dataset. In this dataset, only 27.3% of the surface residues are interface residues. If all surface residues are used in the training, the classifier will be biased to predict a residue as a surface residue. To address this issue, a set of surface residues is randomly selected to make the ratio of positive and negative data 1:1. Fivefold cross-validation is then used to evaluate the SVM. The whole dataset is randomly divided into five subgroups with an approximately equal number of chains. Each SVM runs five times with five different training and test sets. For each run, three of the subsets are used as the training set, one subset is used to select the optimal parameters and the remaining one is used as the test set.

### Performance metrics

The following measures are used to evaluate the performances: precision, recall, accuracy, F1 and correlation coefficient (CC), which are defined as follows:

Precision=TPTP+FP
 MathType@MTEF@5@5@+=feaafiart1ev1aaatCvAUfKttLearuWrP9MDH5MBPbIqV92AaeXatLxBI9gBaebbnrfifHhDYfgasaacH8akY=wiFfYdH8Gipec8Eeeu0xXdbba9frFj0=OqFfea0dXdd9vqai=hGuQ8kuc9pgc9s8qqaq=dirpe0xb9q8qiLsFr0=vr0=vr0dc8meaabaqaciaacaGaaeqabaqabeGadaaakeaacqqGqbaucqqGYbGCcqqGLbqzcqqGJbWycqqGPbqAcqqGZbWCcqqGPbqAcqqGVbWBcqqGUbGBcqGH9aqpdaWcaaqaaiabbsfaujabbcfaqbqaaiabbsfaujabbcfaqjabgUcaRiabbAeagjabbcfaqbaaaaa@41A1@

Recall=TPTP+FN
 MathType@MTEF@5@5@+=feaafiart1ev1aaatCvAUfKttLearuWrP9MDH5MBPbIqV92AaeXatLxBI9gBaebbnrfifHhDYfgasaacH8akY=wiFfYdH8Gipec8Eeeu0xXdbba9frFj0=OqFfea0dXdd9vqai=hGuQ8kuc9pgc9s8qqaq=dirpe0xb9q8qiLsFr0=vr0=vr0dc8meaabaqaciaacaGaaeqabaqabeGadaaakeaacqqGsbGucqqGLbqzcqqGJbWycqqGHbqycqqGSbaBcqqGSbaBcqGH9aqpdaWcaaqaaiabbsfaujabbcfaqbqaaiabbsfaujabbcfaqjabgUcaRiabbAeagjabb6eaobaaaaa@3D56@

F1=2×Precision×RecallPrecision+Recall
 MathType@MTEF@5@5@+=feaafiart1ev1aaatCvAUfKttLearuWrP9MDH5MBPbIqV92AaeXatLxBI9gBaebbnrfifHhDYfgasaacH8akY=wiFfYdH8Gipec8Eeeu0xXdbba9frFj0=OqFfea0dXdd9vqai=hGuQ8kuc9pgc9s8qqaq=dirpe0xb9q8qiLsFr0=vr0=vr0dc8meaabaqaciaacaGaaeqabaqabeGadaaakeaacqqGgbGrcqaIXaqmcqGH9aqpdaWcaaqaaiabikdaYiabgEna0kabbcfaqjabbkhaYjabbwgaLjabbogaJjabbMgaPjabbohaZjabbMgaPjabb+gaVjabb6gaUjabgEna0kabbkfasjabbwgaLjabbogaJjabbggaHjabbYgaSjabbYgaSbqaaiabbcfaqjabbkhaYjabbwgaLjabbogaJjabbMgaPjabbohaZjabbMgaPjabb+gaVjabb6gaUjabgUcaRiabbkfasjabbwgaLjabbogaJjabbggaHjabbYgaSjabbYgaSbaaaaa@5D95@

Accuracy=TP+TNTP+TN+FP+FN
 MathType@MTEF@5@5@+=feaafiart1ev1aaatCvAUfKttLearuWrP9MDH5MBPbIqV92AaeXatLxBI9gBaebbnrfifHhDYfgasaacH8akY=wiFfYdH8Gipec8Eeeu0xXdbba9frFj0=OqFfea0dXdd9vqai=hGuQ8kuc9pgc9s8qqaq=dirpe0xb9q8qiLsFr0=vr0=vr0dc8meaabaqaciaacaGaaeqabaqabeGadaaakeaacqqGbbqqcqqGJbWycqqGJbWycqqG1bqDcqqGYbGCcqqGHbqycqqGJbWycqqG5bqEcqGH9aqpdaWcaaqaaiabbsfaujabbcfaqjabgUcaRiabbsfaujabb6eaobqaaiabbsfaujabbcfaqjabgUcaRiabbsfaujabb6eaojabgUcaRiabbAeagjabbcfaqjabgUcaRiabbAeagjabb6eaobaaaaa@4998@

CC=TP×TN−FP×FN(TP+FN)(TP+FP)(TN+FP)(TN+FN)
 MathType@MTEF@5@5@+=feaafiart1ev1aaatCvAUfKttLearuWrP9MDH5MBPbIqV92AaeXatLxBI9gBaebbnrfifHhDYfgasaacH8akY=wiFfYdH8Gipec8Eeeu0xXdbba9frFj0=OqFfea0dXdd9vqai=hGuQ8kuc9pgc9s8qqaq=dirpe0xb9q8qiLsFr0=vr0=vr0dc8meaabaqaciaacaGaaeqabaqabeGadaaakeaacqWGdbWqcqWGdbWqcqGH9aqpdaWcaaqaaiabbsfaujabbcfaqjabgEna0kabbsfaujabb6eaojabgkHiTiabbAeagjabbcfaqjabgEna0kabbAeagjabb6eaobqaamaakaaabaGaeiikaGIaeeivaqLaeeiuaaLaey4kaSIaeeOrayKaeeOta4KaeiykaKIaeiikaGIaeeivaqLaeeiuaaLaey4kaSIaeeOrayKaeeiuaaLaeiykaKIaeiikaGIaeeivaqLaeeOta4Kaey4kaSIaeeOrayKaeeiuaaLaeiykaKIaeiikaGIaeeivaqLaeeOta4Kaey4kaSIaeeOrayKaeeOta4KaeiykaKcaleqaaaaaaaa@5AAE@

where TP is the number of true positives (interface residues correctly classified as interface residues), FP is the number of false positives (surface residues incorrectly classified as interface residues), TN is the number of true negatives (surface residues correctly classified as surface residues) and FN is the number of false negatives (interface residues incorrectly classified as surface residues).

Precision, recall and F1 are used to measure the performance of classifying interface residues, while accuracy is used to measure the performance of classifying the whole test dataset. Correlation coefficient (CC) is applied to measure the correlation between predictions and actual test data.

## Authors' contributions

QD carried out the binding sites prediction studies, participated in coding and drafted the manuscript. LL and YY participated in the design of the study and performed the statistical analysis. XW conceived of the study, and participated in its design and coordination. All authors read and approved the final manuscript.

## Supplementary Material

Additional file 1 binary profile interface propensities. binary profile interface propensities of hetero-permanent complexes Click here for file

Additional file 2 binary profile interface propensities. binary profile interface propensities of hetero-transient complexes Click here for file

Additional file 3 binary profile interface propensities. binary profile interface propensities of homo-permanent complexes Click here for file

Additional file 4 binary profile interface propensities. binary profile interface propensities of homo-transient complexes Click here for file

## References

[B1] Zhang Z, Grigorov MG (2006). Similarity networks of protein binding sites. Proteins.

[B2] Chelliah V, Chen L, Blundell TL, Lovell SC (2004). Distinguishing structural and functional restraints in evolution in order to identify interaction sites. J Mol Biol.

[B3] Jones S, Thornton JM (1997). Analysis of protein-protein interaction sites using surface patches. J Mol Biol.

[B4] Magliery TJ, Regan L (2005). Sequence variation in ligand binding sites in proteins. BMC Bioinformatics.

[B5] Lo Conte L, Chothia C, Janin J (1999). The atomic structure of protein-protein recognition sites. J Mol Biol.

[B6] Bradford JR, Westhead DR (2005). Improved prediction of protein-protein binding sites using a support vector machines approach. Bioinformatics.

[B7] Nooren IM, Thornton JM (2003). Structural characterisation and functional significance of transient protein-protein interactions. J Mol Biol.

[B8] Bradford JR, Needham CJ, Bulpitt AJ, Westhead DR (2006). Insights into protein-protein interfaces using a Bayesian network prediction method. J Mol Biol.

[B9] Chakrabarti P, Janin J (2002). Dissecting protein-protein recognition sites. Proteins.

[B10] Pils B, Copley RR, Schultz J (2005). Variation in structural location and amino acid conservation of functional sites in protein domain families. BMC Bioinformatics.

[B11] Lichtarge O, Bourne HR, Cohen FE (1996). An evolutionary trace method defines binding surfaces common to protein families. J Mol Biol.

[B12] Morgan DH, Kristensen DM, Mittelman D, Lichtarge O (2006). ET viewer: an application for predicting and visualizing functional sites in protein structures. Bioinformatics.

[B13] Yao H, Kristensen DM, Mihalek I, Sowa ME, Shaw C, Kimmel M, Kavraki L, Lichtarge O (2003). An accurate, sensitive, and scalable method to identify functional sites in protein structures. J Mol Biol.

[B14] Yao H, Mihalek I, Lichtarge O (2006). Rank information: a structure-independent measure of evolutionary trace quality that improves identification of protein functional sites. Proteins.

[B15] Chung JL, Wang W, Bourne PE (2006). Exploiting sequence and structure homologs to identify protein-protein binding sites. Proteins.

[B16] Cheng G, Qian B, Samudrala R, Baker D (2005). Improvement in protein functional site prediction by distinguishing structural and functional constraints on protein family evolution using computational design. Nucleic Acids Res.

[B17] Panchenko AR, Kondrashov F, Bryant S (2004). Prediction of functional sites by analysis of sequence and structure conservation. Protein Sci.

[B18] Valdar WS (2002). Scoring residue conservation. Proteins.

[B19] La D, Sutch B, Livesay DR (2005). Predicting protein functional sites with phylogenetic motifs. Proteins.

[B20] Kim Y, Subramaniam S (2006). Locally defined protein phylogenetic profiles reveal previously missed protein interactions and functional relationships. Proteins.

[B21] Liu AH, Zhang X, Stolovitzky GA, Califano A, Firestein SJ (2003). Motif-based construction of a functional map for mammalian olfactory receptors. Genomics.

[B22] Wang B, Chen P, Huang DS, Li JJ, Lok TM, Lyu MR (2006). Predicting protein interaction sites from residue spatial sequence profile and evolution rate. FEBS Lett.

[B23] Yan C, Dobbs D, Honavar V (2004). A two-stage classifier for identification of protein-protein interface residues. Bioinformatics.

[B24] Bordner AJ, Abagyan R (2005). REVCOM: a robust Bayesian method for evolutionary rate estimation. Bioinformatics.

[B25] Thibert B, Bredesen DE, Del Rio G (2005). Improved prediction of critical residues for protein function based on network and phylogenetic analyses. BMC Bioinformatics.

[B26] Zhou HX, Shan Y (2001). Prediction of protein interaction sites from sequence profile and residue neighbor list. Proteins.

[B27] Meiler J, Baker D (2006). ROSETTALIGAND: protein-small molecule docking with full side-chain flexibility. Proteins.

[B28] Osterberg F, Morris GM, Sanner MF, Olson AJ, Goodsell DS (2002). Automated docking to multiple target structures: incorporation of protein mobility and structural water heterogeneity in AutoDock. Proteins.

[B29] Laurie AT, Jackson RM (2005). Q-SiteFinder: an energy-based method for the prediction of protein-ligand binding sites. Bioinformatics.

[B30] Zhang C, Liu S, Zhu Q, Zhou Y (2005). A knowledge-based energy function for protein-ligand, protein-protein, and protein-DNA complexes. J Med Chem.

[B31] Torrance JW, Bartlett GJ, Porter CT, Thornton JM (2005). Using a library of structural templates to recognise catalytic sites and explore their evolution in homologous families. J Mol Biol.

[B32] Ivanisenko VA, Pintus SS, Grigorovich DA, Kolchanov NA (2005). PDBSite: a database of the 3D structure of protein functional sites. Nucleic Acids Res.

[B33] Wilczynski B, Hvidsten TR, Kryshtafovych A, Tiuryn J, Komorowski J, Fidelis K (2006). Using local gene expression similarities to discover regulatory binding site modules. BMC Bioinformatics.

[B34] Snyder KA, Feldman HJ, Dumontier M, Salama JJ, Hogue CW (2006). Domain-based small molecule binding site annotation. BMC Bioinformatics.

[B35] Neuvirth H, Raz R, Schreiber G (2004). ProMate: a structure based prediction program to identify the location of protein-protein binding sites. J Mol Biol.

[B36] Res I, Mihalek I, Lichtarge O (2005). An evolution based classifier for prediction of protein interfaces without using protein structures. Bioinformatics.

[B37] Yan C, Terribilini M, Wu F, Jernigan RL, Dobbs D, Honavar V (2006). Predicting DNA-binding sites of proteins from amino acid sequence. BMC Bioinformatics.

[B38] Liang S, Zhang C, Liu S, Zhou Y (2006). Protein binding site prediction using an empirical scoring function. Nucleic Acids Res.

[B39] Rossi A, Marti-Renom MA, Sali A (2006). Localization of binding sites in protein structures by optimization of a composite scoring function. Protein Sci.

[B40] Down T, Leong B, Hubbard TJ (2006). A machine learning strategy to identify candidate binding sites in human protein-coding sequence. BMC Bioinformatics.

[B41] Deng H, Chen G, Yang W, Yang JJ (2006). Predicting calcium-binding sites in proteins – a graph theory and geometry approach. Proteins.

[B42] Chen H, Zhou HX (2005). Prediction of interface residues in protein-protein complexes by a consensus neural network method: test against NMR data. Proteins.

[B43] Dubey A, Realff MJ, Lee JH, Bommarius AS (2005). Support vector machines for learning to identify the critical positions of a protein. J Theor Biol.

[B44] Koike A, Takagi T (2004). Prediction of protein-protein interaction sites using support vector machines. Protein Eng Des Sel.

[B45] Li MH, Lin L, Wang XL, Liu T (2007). Protein-protein interaction site prediction based on conditional random fields. Bioinformatics.

[B46] Ofran Y, Rost B (2003). Analysing six types of protein-protein interfaces. J Mol Biol.

[B47] Altschul SF, Madden TL, Schaffer AA, Zhang JH, Zhang Z, Miller W, Lipman DJ (1997). Gapped Blast and Psi-blast: a new generation of protein database search programs. Nucleic Acids Research.

[B48] Dong Q, Wang XL, Lin L, Xu Z (2006). Domain boundary prediction based on profile domain linker propensity index. Comput Biol Chem.

[B49] Dong Qw, Wang Xl, Lin L (2006). Novel knowledge-based mean force potential at the profile level. BMC Bioinformatics.

[B50] Dong QW, Wang XL, Lin L (2007). Protein remote homology detection based on binary profiles. 1st International Conference on Bioinformatics Research and Development BIRD/LNBI.

[B51] Ofran Y, Rost B (2003). Predicted protein-protein interaction sites from local sequence information. FEBS Lett.

[B52] Sander C, Schneider R (1991). Database of homology-derived protein structures and the structural meaning of sequence alignment. Proteins.

[B53] Karlin S, Brocchieri L (1996). Evolutionary conservation of RecA genes in relation to protein structure and function. J Bacteriol.

[B54] Valdar WS, Thornton JM (2001). Protein-protein interfaces: analysis of amino acid conservation in homodimers. Proteins.

[B55] Kouranov A, Xie L, de la Cruz J, Chen L, Westbrook J, Bourne PE, Berman HM (2006). The RCSB PDB information portal for structural genomics. Nucleic Acids Res.

[B56] Bordner AJ, Abagyan R (2005). Statistical analysis and prediction of protein-protein interfaces. Proteins.

[B57] Henrick K, Thornton JM (1998). PQS: a protein quaternary structure file server. Trends Biochem Sci.

[B58] Nooren IM, Thornton JM (2003). Diversity of protein-protein interactions. Embo J.

[B59] Wu CH, Apweiler R, Bairoch A, Natale DA, Barker WC, Boeckmann B, Ferro S, Gasteiger E, Huang H, Lopez R (2006). The Universal Protein Resource (UniProt): an expanding universe of protein information. Nucleic Acids Res.

[B60] Xenarios I, Salwinski L, Duan XJ, Higney P, Kim SM, Eisenberg D (2002). DIP, the Database of Interacting Proteins: a research tool for studying cellular networks of protein interactions. Nucleic Acids Res.

[B61] Kabsch W, Sander C (1983). Dictionary of Secondary structure in Proteins: Pattern Recognition of Hydrogenbonded and Geometrical Features. Biopolymers.

[B62] Vapnik VN (1998). Statistical learning theory.

[B63] Chang CC, Lin CJ (2001). LIBSVM: a library for support vector machines. http://www.csie.ntu.edu.tw/~cjlin/libsvm.

